# Hip Osteoarthritis Associated with Prominent Synovitis Revealed by ^68^Ga-FAPI PET/CT

**DOI:** 10.3390/diagnostics16142278

**Published:** 2026-07-21

**Authors:** Yiqun Wang, Ruimin Wang, La Li, Wei Rong, Ligong Fu

**Affiliations:** 1 Orthopaedic and Sports Medicine Center, Beijing Tsinghua Changgung Hospital, Tsinghua University, Beijing 100000, China; 2 Department of Nuclear Medicine, The First Medical Centre, Chinese PLA General Hospital, Beijing 100000, China; 3 Department of Sports Medicine, Peking University Third Hospital, Institute of Sports Medicine of Peking University, Beijing Key Laboratory of Sports Injuries, Beijing 100000, China

**Keywords:** osteoarthritis, hip, synovium, FAPI, PET/CT

## Abstract

A 56-year-old patient was initially diagnosed with aseptic loosening of a left acetabular prosthesis based on ^68^Ga-FAPI. Half a year after revision, this patient developed right hip pain and a subsequent ^68^Ga-FAPI was performed revealing increased tracer uptake in the right hip, leading to a diagnosis of synovitis-induced osteoarthritis. This case highlights the utility of ^68^Ga-FAPI-PET/CT in visualizing synovial activity and underscores the potential role of synovitis in the pathogenesis of osteoarthritis.

**Figure 1:**
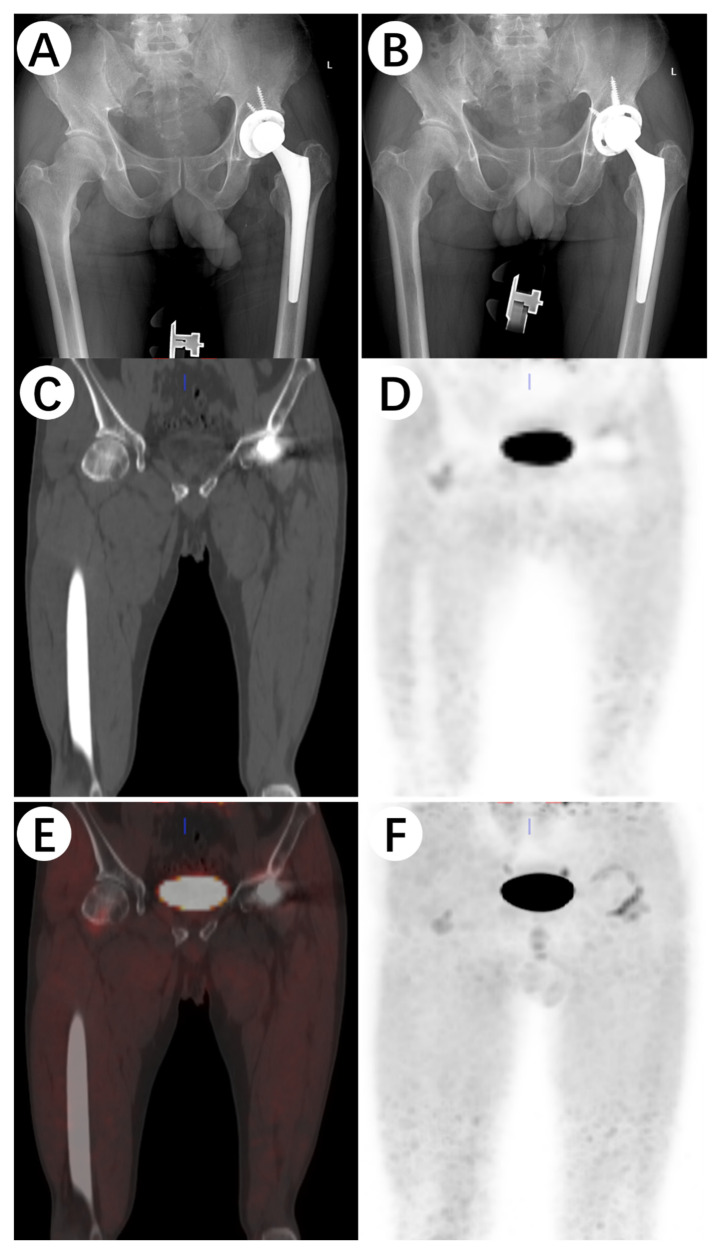
A 56-year-old male patient presented with persistent left hip pain for approximately one year following a total hip arthroplasty (THA), which had been performed following a femoral neck fracture 3.5 years ago. Initial X-ray (**A**) demonstrated that the abduction and anteversion angles seemed not ideal. No obvious joint space narrowing or osteophyte formation was observed in the contralateral (right) hip, corresponding to a Kellgren–Lawrence (K–L) grade of 2. The patient subsequently underwent ^68^Ga-FAPI PET/CT (uMI 510, United Imaging Healthcare, Shanghai, China). Patients were not required to fast before imaging. PET/CT was performed 1 h after intravenous administration of ^68^Ga-FAPI (1.8–2.4 MBq/kg), with an acquisition time of 4 min per bed position. Low-dose CT (120 kV, 30–50 mAs) was used for attenuation correction and anatomical localization, and PET images were reconstructed using a standard ordered-subset expectation maximization algorithm. Through ^68^Ga-FAPI PET/CT (this test was approved by the Ethics Committee of our hospital and registered at the Chinese Trial Registry: ChiCTR2000041204, and written informed consent was obtained from the patient for the purpose of scientific research), obvious linear uptake along the acetabular prosthesis and high uptake at the junction of the head and neck were observed (**C**–**F**). According to our previous report about the diagnostic classification of ^68^Ga-FAPI in pain after hip replacement, this patient was diagnosed with acetabular prosthesis loosening [1], and during revision surgery, the acetabular prosthesis was not stable and replaced. Notably, before revision surgery, mildly increased ^68^Ga-FAPI uptake was already present at the right femoral head–neck junction (SUVmax = 3.87), despite the absence of corresponding radiographic abnormalities. Half a year after revision, the patient returned to our hospital and complained about severe pain in the right hip. Follow-up X-ray (**B**) again showed no definite joint space narrowing or osteophyte formation, corresponding to a K–L grade of 2; however, subtle surface irregularity of the right femoral head cartilage was observed compared with the pre-revision radiograph. Initial laboratory tests were mildly abnormal, with the results of C-reactive protein (mg/dL) and ESR (mm/h) being 1.272 and 69, respectively.

**Figure 2:**
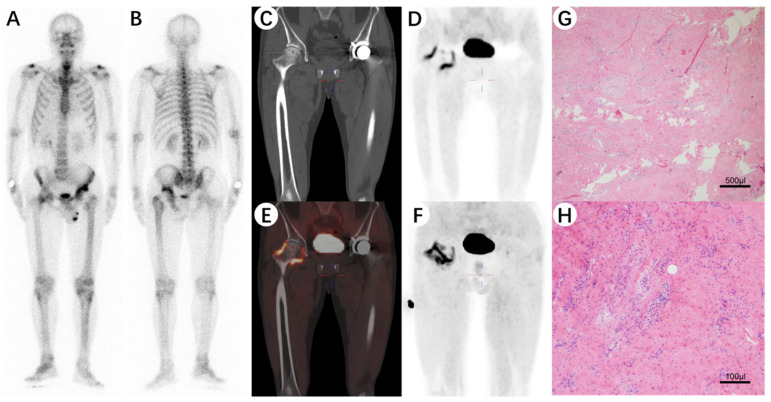
CT (**C**) revealed bone cyst formation in the weight-bearing area of the femoral head. To further confirm the diagnosis, bone scans, MRI (uMR 570, United Imaging Healthcare, Shanghai, China) and ^68^Ga-FAPI PET/CT were performed. The bone scan (**A**,**B**) showed high uptake in the left acetabulum (due to bone remodeling) and moderate uptake in the femoral neck. MRI of the right hip showed no significant bone marrow edema or joint swelling, with mild synovial hyperplasia. While the result of ^68^Ga-FAPI PET/CT (**C**–**F**) was interesting, the synovium around the femoral neck showed intense high uptake (SUVmax: 19.17). Despite the abnormal serological results, the collective evidence from previous medical history, imaging examination, synovial fluid culture, synovial fluid leukocyte count and neutrophil percentage did not support the diagnosis of septic arthritis. The patient was therefore diagnosed with hip osteoarthritis associated with marked synovitis and subsequently underwent right total hip arthroplasty. During surgery, the frozen section demonstrated focal hyaline degeneration with mild fatty infiltration, accompanied by fibroblast proliferation and extracellular matrix deposition, whereas no neutrophil infiltration was identified (**G**,**H**), and no sign of infection was observed in the operation area. Postoperative tissue cultures from three independent specimens remained negative after 21 days of incubation following a 14-day antibiotic-free interval. The final histopathological findings were consistent with the intraoperative frozen-section diagnosis. One year follow-up, the patient reported substantial symptomatic improvement, with a visual analog scale pain score of 1 and a Harris Hip Score of 90. Inflammatory markers, including C-reactive protein, erythrocyte sedimentation rate, and white blood cell count, had all returned to normal. Osteoarthritis is a prevalent and clinically challenging condition in orthopedics, and its pathogenesis is usually considered to be caused by the disruption of articular cartilage [2,3,4]. Current understanding, however, defines osteoarthritis as a whole-joint disease, characterized by pathological changes not only in articular cartilage but also in the synovium, joint capsule, and periarticular soft tissues [5,6]. The precise initiating event—whether it originates in the cartilage or the synovium—remains a subject of debate, analogous to the “chicken-and-egg” dilemma. More importantly, OA is now recognized as a heterogeneous disease comprising several distinct phenotypes, including genetic, post-traumatic, aging-related, metabolic, hormonal, mechanical overload, and inflammatory subtypes [7]. In this study, following revision arthroplasty on the left side, the patient relied predominantly on the right lower limb for weight bearing. This clinical course resembles the commonly observed phenomenon in which patients initially develop unilateral joint pain and subsequently experience symptoms in the contralateral joint after prolonged compensatory loading. Although a causal relationship cannot be established from a single case, mechanical overload may have contributed to synovial activation before irreversible structural degeneration became apparent. This interpretation is consistent with the concept that alterations in the biological microenvironment may precede detectable anatomical abnormalities. Recent studies have also shown that the synovium, as a giant cytokine pool, could accelerate the progression of osteoarthritis and that FAP could reflect the synovial state [8,9]. The present case illustrates this concept particularly well. Conventional radiography demonstrated only minimal degenerative changes, corresponding to Kellgren–Lawrence grade 2, while MRI failed to reveal characteristic abnormalities. In contrast, ^68^Ga-FAPI PET/CT demonstrated intense tracer uptake within the synovium. These findings suggest that synovial activation may become detectable before substantial structural alterations are apparent on conventional imaging. An additional observation deserves particular attention. Retrospective review of the patient’s initial ^68^Ga-FAPI PET/CT revealed mild but definite tracer uptake at the femoral head–neck junction. Although this finding was not considered clinically significant at that time, it became particularly meaningful in light of the patient’s subsequent clinical course. Whether this early molecular abnormality represented the initial stage of disease progression cannot be determined from a single case. Nevertheless, it raises the possibility that ^68^Ga-FAPI PET/CT may identify biologically active joint lesions before irreversible structural damage becomes established. In summary, although FAP is a spectrum-associated protein that is upregulated in a variety of diseases, this case demonstrates that ^68^Ga-FAPI PET/CT can visualize prominent synovial fibroblast activity in a clinically challenging case of hip osteoarthritis, providing molecular information beyond that available from conventional imaging modalities. These findings further support the emerging concept that synovial activation may play an important role in the early pathogenesis of osteoarthritis. For patients with clinically suspected osteoarthritis but inconclusive findings on conventional imaging, ^68^Ga-FAPI PET/CT may serve as a valuable complementary imaging modality by facilitating the detection of early biological changes. Although the present observations are limited to a single case, they highlight the potential clinical utility of FAP-targeted molecular imaging and provide a rationale for further investigation into the role of synovial FAP expression in the pathogenesis, early diagnosis, and therapeutic monitoring of osteoarthritis.

## Data Availability

The datasets generatedduring the current article are available from the corresponding author on reasonable request.
